# *Amblyomma americanum* ticks utilizes countervailing pro and anti-inflammatory proteins to evade host defense

**DOI:** 10.1371/journal.ppat.1008128

**Published:** 2019-11-22

**Authors:** Mariam Bakshi, Tae Kwon Kim, Lindsay Porter, Waithaka Mwangi, Albert Mulenga

**Affiliations:** 1 Department of Veterinary Pathobiology, College of Veterinary Medicine, TAMU, College Station, Texas, United States of America; 2 Department of Diagnostic Medicine/Pathobiology, College of Veterinary Medicine, Kansas State University, Manhattan, Kansas, United States of America; University of South Alabama, UNITED STATES

## Abstract

Feeding and transmission of tick-borne disease (TBD) agents by ticks are facilitated by tick saliva proteins (TSP). Thus, defining functional roles of TSPs in tick evasion is expected to reveal potential targets in tick-antigen based vaccines to prevent TBD infections. This study describes two types of *Amblyomma americanum* TSPs: those that are similar to LPS activate macrophage (MΦ) to express pro-inflammation (PI) markers and another set that suppresses PI marker expression by activated MΦ. We show that similar to LPS, three recombinant (r) *A*. *americanum* insulin-like growth factor binding-related proteins (r*Aam*IGFBP-rP1, r*Aam*IGFBP-rP6S, and r*Aam*IGFBP-rP6L), hereafter designated as PI-rTSPs, stimulated both PBMC -derived MΦ and mice RAW 267.4 MΦ to express PI co-stimulatory markers, CD40, CD80, and CD86 and cytokines, TNFα, IL-1, and IL-6. In contrast, two *A*. *americanum* tick saliva serine protease inhibitors (serpins), AAS27 and AAS41, hereafter designated as anti-inflammatory (AI) rTSPs, on their own did not affect MΦ function or suppress expression of PI markers, but enhanced expression of AI cytokines (IL-10 and TGFβ) in MΦ that were pre-activated by LPS or PI-rTSPs. Mice paw edema test demonstrated that *in vitro* validated PI- and AI-rTSPs are functional *in vivo* since injection of HEK293-expressed PI-rTSPs (individually or as a cocktail) induced edema comparable to carrageenan-induced edema and was characterized by upregulation of CD40, CD80, CD86, TNF-α, IL-1, IL-6, and chemokines: CXCL1, CCL2, CCL3, CCL5, and CCL11, whereas the AI-rTSPs (individually and cocktail) were suppressive. We propose that the tick may utilize countervailing PI and AI TSPs to regulate evasion of host immune defenses whereby TSPs such as r*Aam*IGFBP-rPs activate host immune cells and proteins such as AAS27 and AAS41 suppress the activated immune cells.

## Author summary

Several studies have documented immuno-suppressive activities in whole tick saliva and salivary gland protein extracts. We have made contribution toward understanding the molecular basis of tick feeding, as we have described functions of defined tick saliva immuno-modulatory proteins. We have shown that *A*. *americanum* injects two groups of functionally opposed tick saliva proteins: those that could counter-intuitively be characterized as pro-host defense, and those that are expected to have anti-host immune defense functions. Based on our data, we propose that the tick evades host defense using countervailing pro- and anti- inflammatory proteins in which the pro-host defense tick saliva proteins stimulate host immune cells such as macrophages, and the anti-host defense tick saliva proteins suppress functions of the activated immune cells.

## Introduction

Ticks are among the most important ecto-parasites with global public and veterinary health impact. In terms of diversity of transmitted disease pathogens, ticks far outpace any known vector arthropod, and are considered second to mosquitoes in terms of impact of the transmitted disease pathogens. In the livestock industry, losses due to ticks and tick-borne diseases (TBD) are estimated to be worth millions of US dollars annually [1]. Globally, the impact of TBD in public health has been on the rise, with the food-for-thought article on “One Health” listing several TBDs among sources of human health concerns needing One Health solutions [2]. Similarly, of the 23 human vector-borne diseases (VBD) that were listed by the World Health Organization, seven are TBD agents: Crimean-Congo hemorrhagic fever, Lyme disease, relapsing fever (borreliosis), rickettsial diseases (spotted fever and Q fever), tick-borne encephalitis, and tularemia (http://www.who.int/mediacentre/factsheets/fs387/en/). In the United States, the Centers for Disease Control (CDC) listed 16 human TBD agents (http://www.cdc.gov/ticks/diseases), six of which were transmitted by *Ixodes scapularis* and four by *Amblyomma americanum* ticks. Additionally, six human TBD agents were listed on the 2018 National Notifiable human VBD in the USA and its territories. In fact, from 2004 to 2016, the six human TBDs accounted for nearly 77% of human VBDs in the USA and its territories [3].

In the absence of effective vaccines against major TBD agents, controlling ticks using acaricides remains the only method to protect animals and humans against TBD infections [4]. Serious limitations such as environmental contamination and ticks developing resistance that threaten acaricide-based tick control have justified the need to develop alternative tick control methods [5–8]. Immunization of animals against tick feeding has been validated as an alternative method [9]. The approach is attractive because it is environmentally friendly and is postulated to be effective against both susceptible and acaricide-resistant tick populations. The limiting step is the availability of effective vaccine antigens. Except for a few instances when human TBD infections occurred after exposure to contaminated materials [10–16], transmission of both animal and human TBD agents occur during tick feeding. Thus, a deeper understanding of how the tick accomplishes feeding is a rational approach through which vaccine targets can be identified [17–21].

Host inflammatory response is the first line of defense against the tick feeding style of disrupting host tissue and sucking up blood. Macrophages (MΦ), which have been confirmed to infiltrate the tick feeding site [22–25] are among key effector cells of the inflammatory response. Importantly, MΦ act as a bridge between innate and adaptive immunity [26, 27]. From this perspective, it is logical that parasites including ticks might target MΦ to evade host immune defenses. A limited number of studies have documented immuno-modulatory effects against MΦ functions by mostly yet undefined tick salivary factors. Salivary gland protein extracts (SGE) of fully fed *Rhipicephalus microplus* suppressed expression of pro-inflammatory (PI) co-stimulatory markers CD86 and CD69, but not CD40 and CD80 by LPS-activated MΦ (92). The same authors found that *R*. *microplus* SGE suppressed secretion of PI cytokines by LPS activated MΦ [28]. Likewise, SGE of *D*. *variabilis* inhibited secretion of PI cytokines by LPS-activated MΦ [29]. In this same study, *D*. *variabilis* tick saliva proteins stimulated MΦ to secrete copious amounts of prostaglandin 2, which in turn stimulated fibroblast migration. In a related study, *D*. *variabilis* tick saliva was shown to increase both basal and platelet-derived growth factor stimulated migration of MΦ [30]. In another study, *Amblyomma variegatum* tick saliva proteins were shown to suppress expression of MHC-II, CD40, CD80, IL-12-p40, and TNF-α, but increased IL-10 expression by MΦ [31]. Similar immuno-modulatory effects against MΦ functions were also reported in *Rhipicephalus sanguineus* [32]. These studies clearly indicated that ticks target MΦ functions in their quest to evade host immune defenses and facilitate feeding and transmission of TBD agents.

At the time of drafting this manuscript few defined tick proteins that modulated MΦ functions were reported including a pro-inflammatory MΦ migration inhibitory factor has been described in ticks including *A*. *americanum* [33–36], but function was not validated. In another study, *Ixodes scapularis* cystatin was shown to affect MΦ function in response to *Anaplasma phagocytophilum* infection [37]. Here, we report that similar to LPS, *A*. *americanum* insulin-like growth factor binding protein-related proteins (*Aam*IGFBP-rP1, *Aam*IGFBP-rP6L, and *Aam*IGFBP-rP6S) [38, 39] stimulated MΦ to express PI markers. Interestingly, we also show that *A*. *americanum* serine protease (AAS) inhibitors (serpin)-27 and 41 [40], blocked LPS and *Aam*IGFBP-rPs activated MΦ to express PI markers. We have described our findings with reference to understanding molecular mechanisms that regulate tick feeding.

## Results

### *Amblyomma americanum* insulin-like growth factors (r*Aam*IGFBP-rP1, r*Aam*IGFBP-rP6S, and r*Aam*IGFBP-rP6L) induced pro-inflammatory response in macrophages (MΦ)

**Fig 1:**
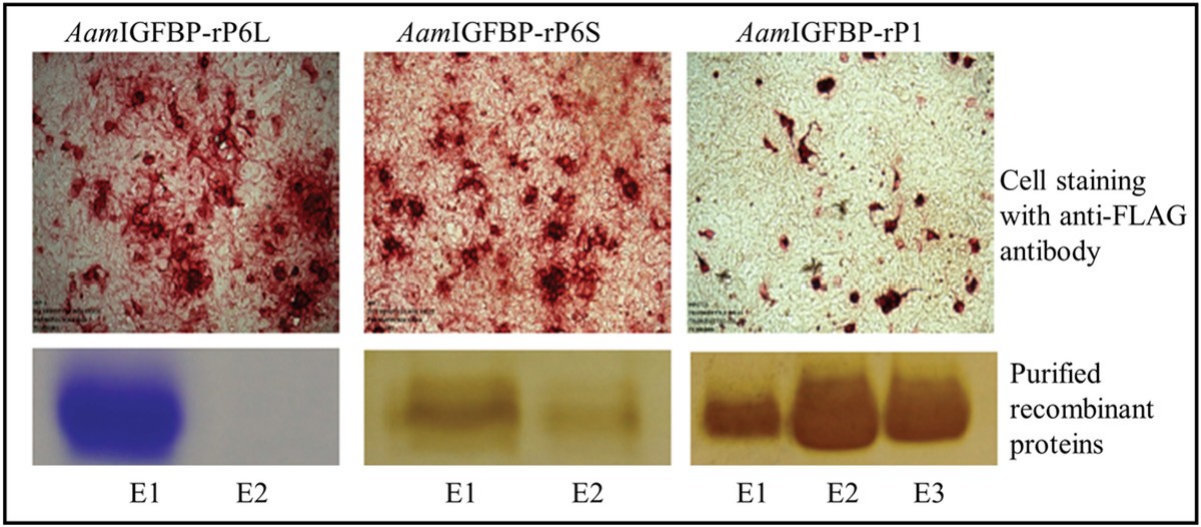
Expression of recombinant *Amblyomma americanum* tick saliva insulin-like growth factor binding proteins-related proteins (*Aam*IGFBP-rP) in Human Embryonic Kidney (HEK) 293 cells. Top panel: HEK cells expressing recombinant tick saliva proteins were immune-stained using the antibody to the FLAG tag as indicated by red staining. Bottom panel: Coomassie or silver stained of affinity purified r*Aam*IGFBP-rPL, r*Aam*IGFBP-rP6S, and r*Aam*IGFBP-rP1 elutions (E) on 12.5% acrylamide gels.

**Fig 2:**
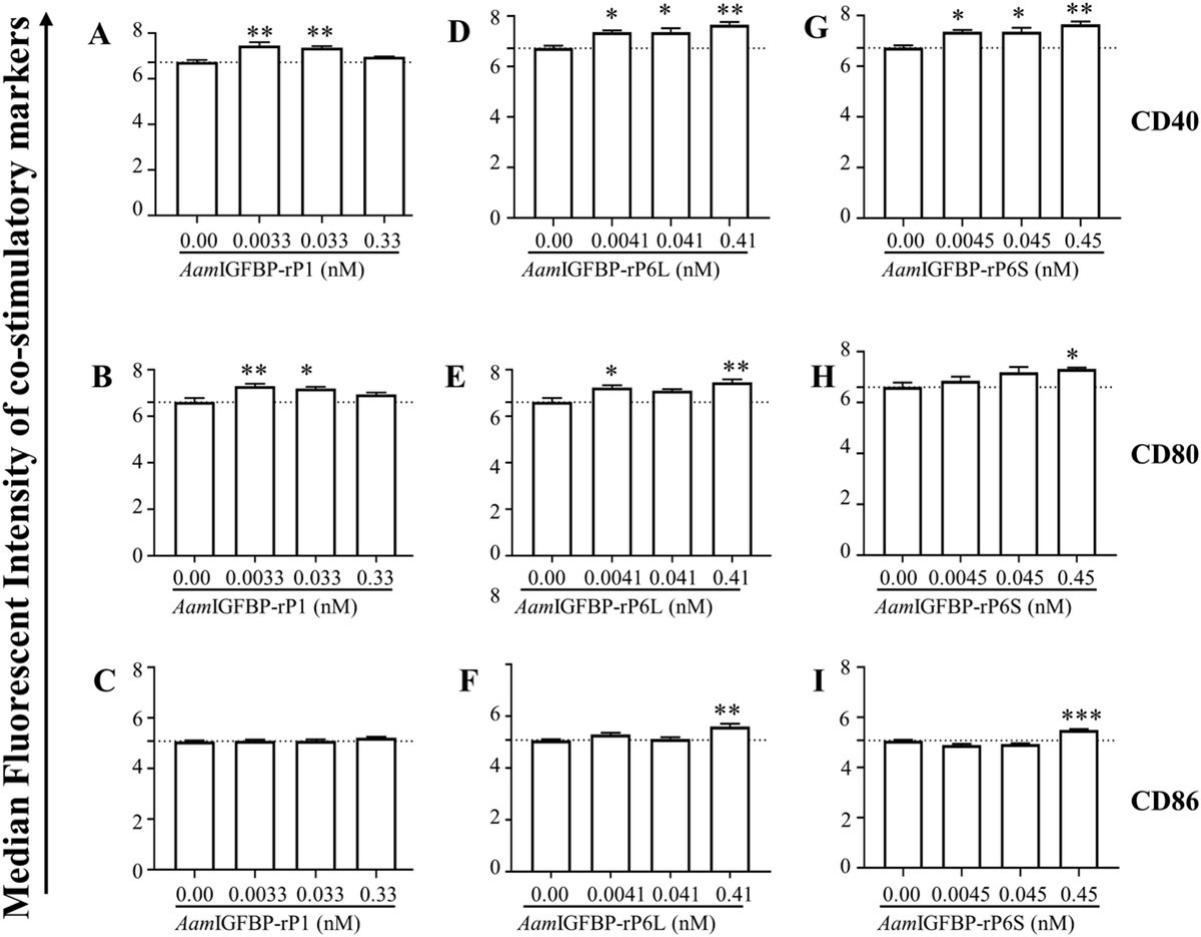
HEK 293 cell-expressed r*Aam*IGFBP-rPs stimulated MΦ to express pro-inflammatory co-stimulatory markers and nitric oxide (NO) secretion. Median fluorescent intensities of co-stimulatory markers CD40 (Fig 2A, 2D and 2G), CD80 Fig 2B, 2E and 2H), and CD86 (Fig 2C, 2F and 2I) in RAW MΦ 264.7 that were treated with increasing amount of r*Aam*IGFBP-rP1, r*Aam*IGFBP-rP6L and r*Aam*IGFBP-rP6S. Data are reported as the mean (three biological replicates) ± SE of three replicates. (*) = p ≤ 0.05, (**) = p ≤ 0.01, (***) = p ≤ 0.001, (****) = p ≤ 0.0001, indicating statistically significant difference between media and treatments. No asterisks indicated represent non-statistical significance.

Flow cytometric analyses was used to demonstrate that similar to LPS, [39] HEK293-expressed r*Aam*IGFBP-rP1, r*Aam*IGFBP-rP6S, and r*Aam*IGFBP-rP6L (Fig 1) stimulated mice RAW 267 MΦ to significantly express PI co-stimulatory markers, CD40, CD80, and CD86 (Fig 2).

Low (0.1 μg/ml) and medium (1 μg/mL) of doses of r*Aam*IGFBP-rP1 induced significant expression of CD40 and CD80 (Fig 2A and 2B) but not CD86 (Fig 2C). For *Aam*IGFBP-rP6L, all three doses induced high expression of CD40 (Fig 2D), CD80 by the low and high dose (Fig 2E), and CD86 by high dose (Fig 2F). Similarly, all doses of r*Aam*IGFBP-rP6S induced significant expression of CD40 (Fig 2G), while significant expression of both CD80 and CD86 were observed at the high dose (Fig 2H and 2I).

**Fig 3:**
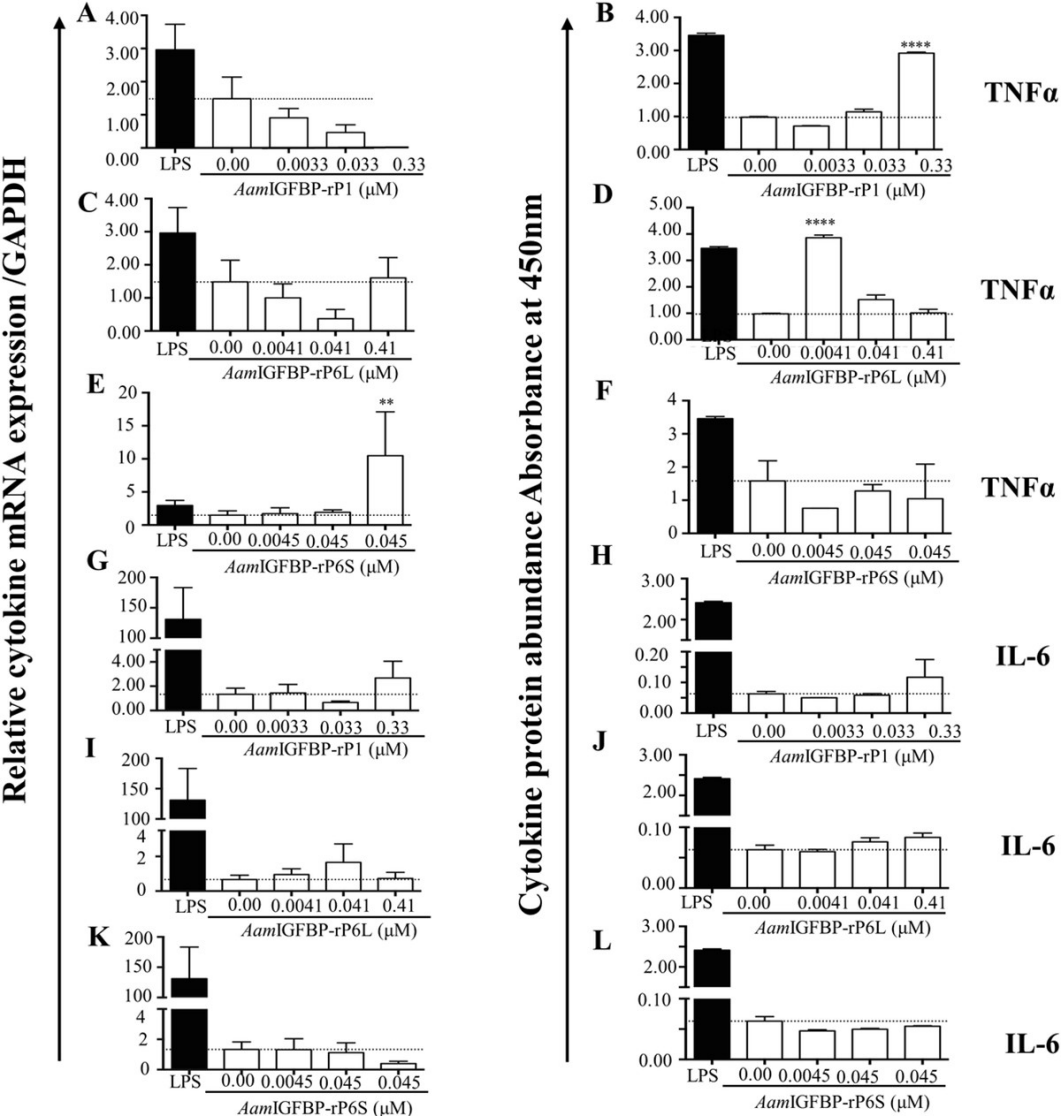
HEK expressed r*Aam*IGFBP-rPs stimulated MΦ to express pro-inflammation cytokines. (A, C, E, G, I, K) Relative transcript abundance and (B, D, F, H, J, L) Secretion abundance of TNFα, IL-6 and IL-1 in cell culture supernatants from MΦ that were treated with various doses of r*Aam*IGFBP-rP1, r*Aam*IGFBP-rP6L and r*Aam*IGFBP-rP6S. Data are reported as the mean (three biological replicates) absorbance values ± SE of three replicates. (**) = p ≤ 0.01, (***) = p ≤ 0.001, (****) = p ≤ 0.0001, indicating statistically significant difference between media and treatments. No asterisks indicated represent non-statistical significance.

We further analyzed the effects on MΦ expression of PI cytokines (Fig 3). Furthermore, ELISA results determined the expression of PI cytokines showed that r*Aam*IGFBP-rP1- and r*Aam*IGFBP-rP6L- treated MΦ induced expression of TNF-α and IL-6 (Fig 3). The effects of r*Aam*IGFBP-rP1 and *Aam*IGFBP-rP6L displayed a dichotomous effect on TNFα expression: whereas transcription was not affected (Fig 3A and 3C), it was secreted at significant levels in high dose r*Aam*IGFBP-rP1 (Fig 3B) and low dose r*Aam*IGFBP-rP6L (Fig 3D). In r*Aam*IGFBP-rP6S treated MΦ, the effect was opposite: the high dose induced significant TNF-α transcript but had no effect on its secretion (Fig 3F). In the case of IL-6, both r*Aam*IGFBP-rP1 and *Aam*IGFBP-rP6L apparently induced transcription and secretion (Fig 3G, 3H, 3I and 3J), while r*Aam*IGFBP-rP6S had not effect (Fig 3K and 3L).

**Fig 4:**
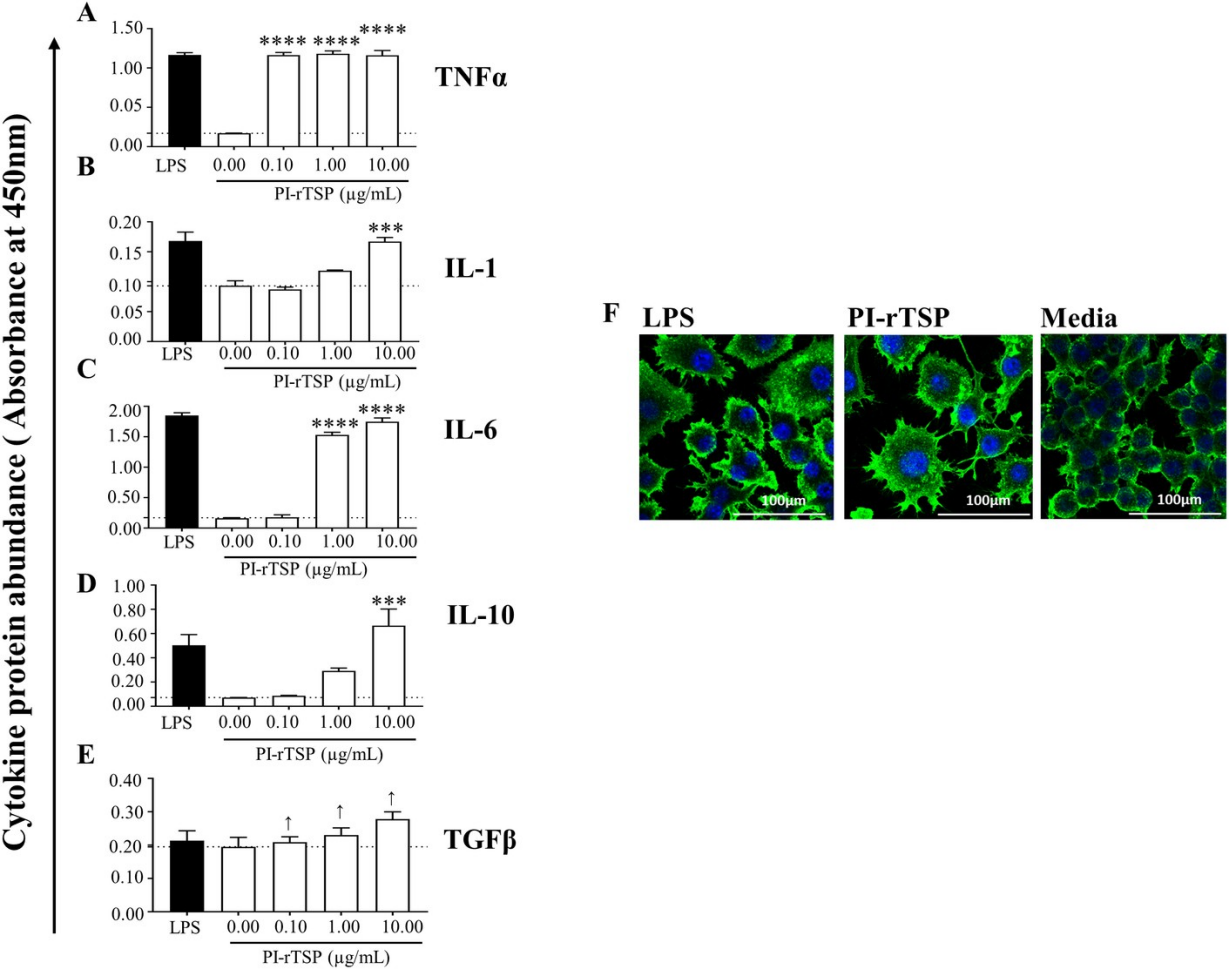
The cocktail of r*Aam*IGFBP-rP1, r*Aam*IGFBP-rP6S, and r*Aam*IGFBP-rPL6L synergistically activates MΦ to express pro-inflammation cytokines and anti-inflammation cytokines similar to LPS. (A-E) Secretion abundance of IL-1, IL-6, IL-10 and TGFβ and (F) Microscopic examination of RAW macrophages 264.7 (MΦ) that were treated with r*Aam*IGFBP-rP1, r*Aam*IGFBP-rP6S, and r*Aam*IGFBP-rPL6L cocktail. Data are reported as the mean of three biological replicates: *A*_450nm_ values ± SEM. Asterisks (*) = p ≤ 0.05, (**) = p ≤ 0.01, (***) = p ≤ 0.001, (****) = p ≤ 0.0001 indicating statistically significant difference between media and treatments. No asterisks indicated represent non-statistical significance.

We next tested the synergistic effect of the three PI-rTSPs as a cocktail (Fig 4). It is interesting to note that secretion levels of TNF-α, IL-1, and IL-6, were statistically similar to levels that were induced by LPS activated of MΦ (Fig 4A, 4B and 4C). Treatment of MΦ with the PI-rTSP cocktail might have over activated the cells in that AI cytokines, IL-10 (Fig 4D) and TGFβ (Fig 4E) were also significantly induced.

Next, we tested the effects of PI-rTSPs cocktail on MΦ morphology (Fig 4F). Confocal microscopy revealed that PI-rTSPs activated MΦ had the morphology that was similar to LPS activated MΦ as indicated by stretched spindle-like shaped cells compared to smooth and round cells in the media only control (Fig 4F). Altogether, results from the *in vitro* study confirmed that the PI-rTSPs stimulated MΦ to express PI markers.

### AAS27 and AAS41 suppressed expression of pro-inflammatory, but enhanced anti-inflammatory markers by LPS- and PI-rTSPs-activated MΦ

**Fig 5:**
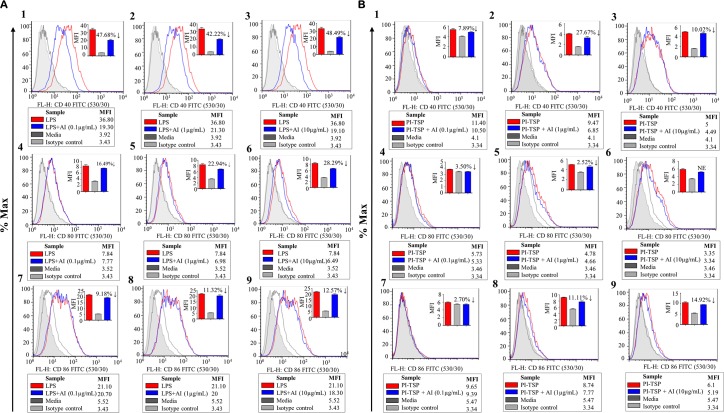
*A*. *americanum* serpin (AAS) 27 and 41 reverses expression of co-stimulatory markers by LPS or r*Aam*IGFBP-rP activated macrophages. (5A, 1–9) Median fluorescent intensity levels of co-stimulatory markers CD40, CD80 and CD86, on RAW MΦ treated with LPS or (5B, 1–9) with various doses of PI-rTSPs followed by AAS27 and AAS41 cocktail. Filled gray chromatogram = isotype control, dark gray bar graph = control (media treated cells); Blue line chromatogram and bar = MΦ that were pre-activated by LPS or PI-rTSP followed by treatment with AAS27 and AAS41 cocktail; Red line chromatogram and bar = MΦ treated with LPS or PI-rTSP only. Please note that, histograms are representative of one treatment. Graph inserts are mean of three biological replicates and presented as absorbance values ± SEM. Arrows (↓) indicate percentage (%) decrease in CD40, CD80 or CD86 expression.

Preliminary findings suggested that rAAS27 and rAAS41 on their own did not affect MΦ function but might have affected functions of activated cells. Flow cytometric analyses showed that the rAAS27 and rAAS41 cocktail rTSPs (0.1, 1 and 10μg/mL) respectively suppressed the expression of CD40, CD80, and CD86 by 43–48%, 16–28%, and 9–13% by MΦ that were pre-activated with LPS (Fig 5A1-9). Likewise, though moderately and not significant, AI-rTSP respectively suppressed expression of CD40, CD80, and CD86 by ~7–10%, ~2–3%, and 2–15% by PI-rTSP activated MΦ (Fig 5B1-9).

**Fig 6:**
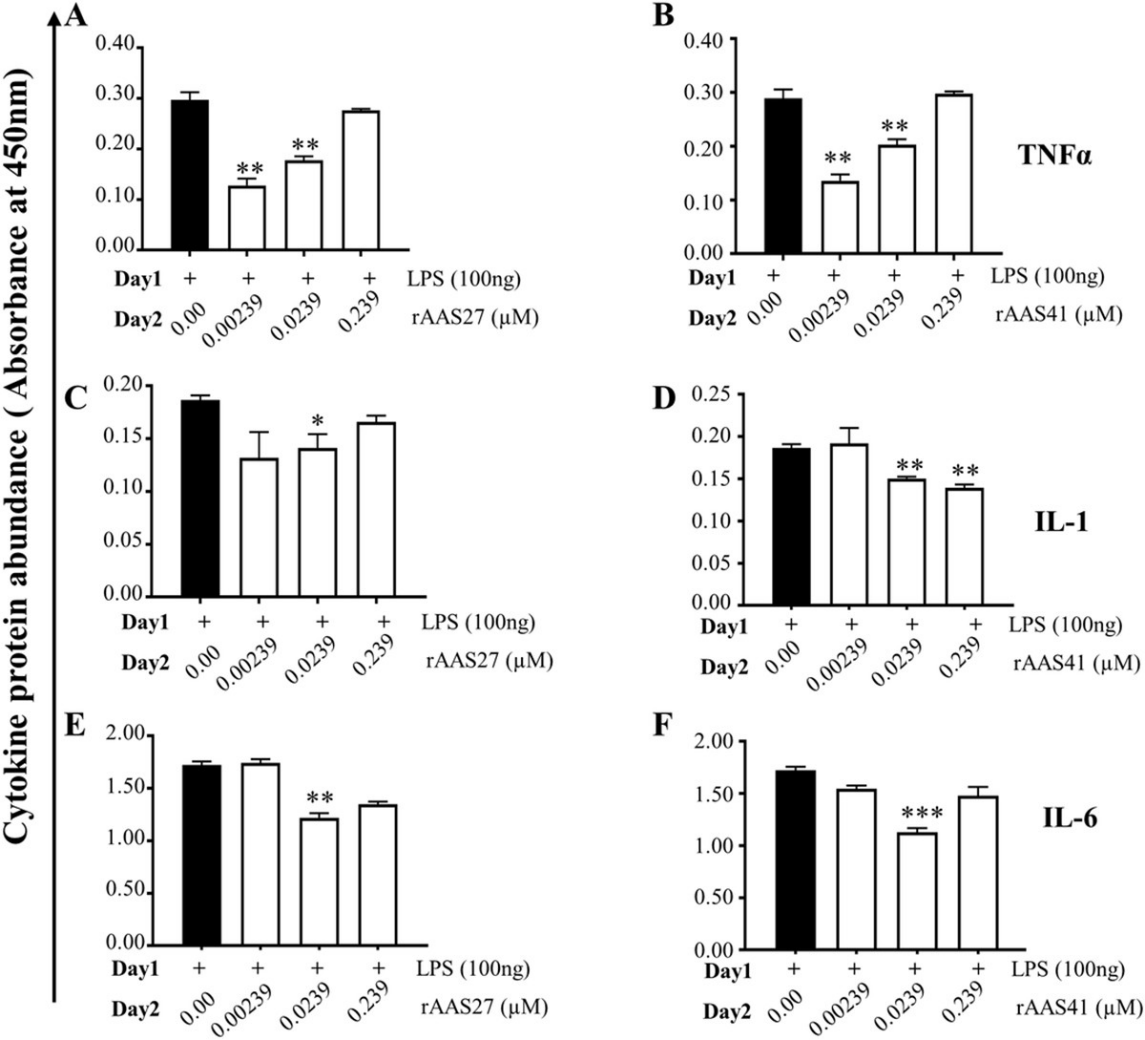
Both AAS27 and AAS41 suppresses expression of pro-inflammation cytokines by LPS activated MΦ. Cytokine (TNFα, IL-1, IL-6) levels in MΦ that were pre-activated with LPS followed by treatment with rAAS27 (A, C, and E) and rAAS41 (B, D, and F) are reported as mean of three biological replicates and reports as *A*_450nm_ values ± SEM. Day 1 = 24 h incubation with 100 ng LPS; Day 2 = Following 24 h with LPS, removal of media and 24 h incubation with 0.0023, 0.023 and 0.23 μM rAAS27 or rAAS41. Asterisks (*) = p ≤ 0.05, (**) = p ≤ 0.01, (***) = p ≤ 0.001, (****) = p ≤ 0.0001, indicating statistically significant difference between media and treatments. No asterisks indicated represent non-statistical significance.

We next analyzed cytokine secretion and ELISA revealed that both rAAS27 and rAAS41 suppressed cytokine expression by LPS-activated MΦ (Fig 6).Low (0.1 μg/ml) and medium dose (1 μg/ml) of rAAS27 and rAAS41 significantly suppressed TNF-α secretion by 30–37% and 52–57% respectively (Fig 6A and 6B). Likewise, the medium (1 μg/ml) dose of rAAS27 suppressed secretion of IL-1 (Fig 6C) and IL-6 (Fig 6E) by 24.85% and 18% respectively (Fig 6E). Similarly, middle and high dose of rAAS41 significantly reduced secretion of IL-1 by up to ~29.32% (Fig 6D) and IL-6 by up to 21.63% (Fig 6F).

**Fig 7:**
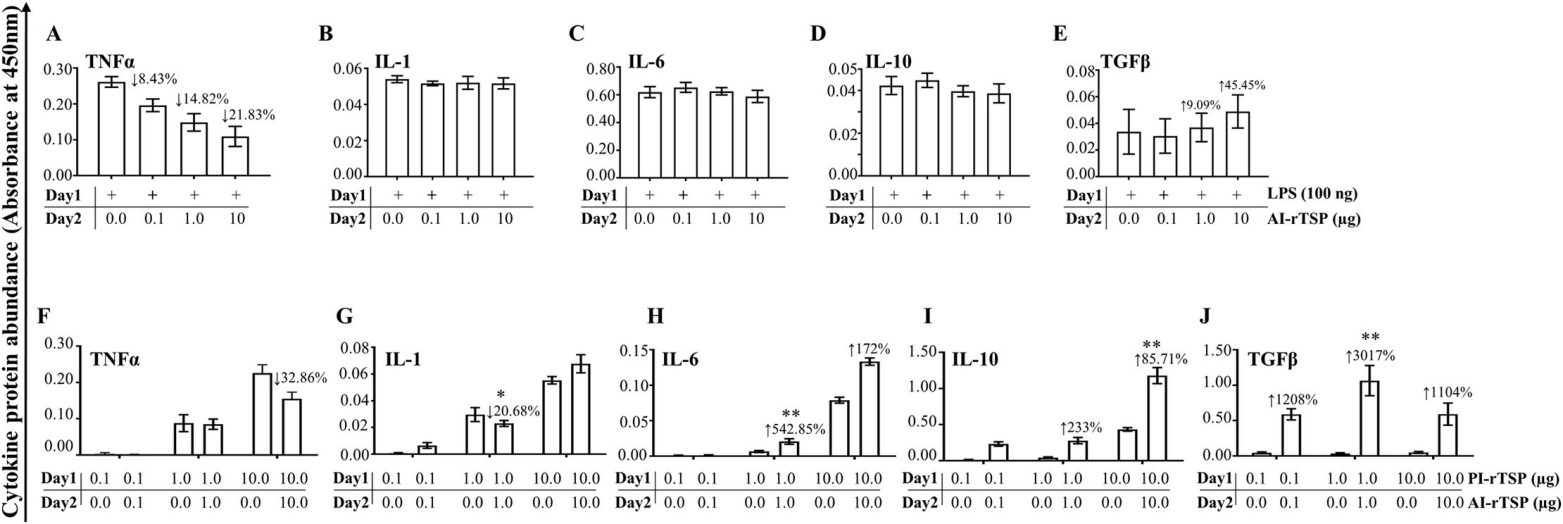
Effect of AAS27 and AAS41 cocktail on expression of pro-inflammatory and anti-inflammatory cytokines by MΦ that were first activated with LPS or cocktail of r*Aam*IGFBP-rP1, r*Aam*IGFBP-rP6S, and r*Aam*IGFBP-rPL6. Cytokines (TNFα, IL-1, IL-6, TGFβ and IL-10) in MΦ that were first activated with LPS (A, B, C, D, and E) or PI-rTSPs (F, G, H, I, and J) followed by treatment with various doses of AAS27 and AAS41 cocktail. Data are reported as mean *A*_450nm_ of two biological replicates ± SEM. Day 1 = 24 h incubation with 100 ng LPS or PI-rTSPs cocktail; Day 2 = Following 24 h with LPS or PI-rTSPs cocktail, removal of media and additional 24 h incubation with AI-rTSPs cocktail. Arrows (↑) or (↓) indicate increase or decrease in cytokine protein secretion compared to LPS control and PI-rTSPs treated MΦ.

We next investigated the effects of the AI-rTSP cocktail on expression of cytokines by MΦ that were first activated by LPS or PI-rTSPs (Fig 7). Except for TNFα, which was apparently suppressed, but not significantly (Fig 7A), there was no effect on IL-1, IL-6, and IL-10 expression by LPS-activated MΦ (Fig 7B, 7C and 7D). In Fig 7E, the AI-rTSP cocktail moderately but not significantly enhanced secretion of TGFβ in LPS activated MΦ. With exception of TNF-α, which was significantly suppressed by the highest dose (Fig 7F), expression of IL-1, IL-6, IL-10, and TGFβ were enhanced when PI-rTSP activated MΦ were treated with the AI-rTSP cocktail (Fig 7G, 7H, 7I and 7J).

### PI-rTSPs and AI-rTSPs are functional *in vivo*

**Fig 8:**
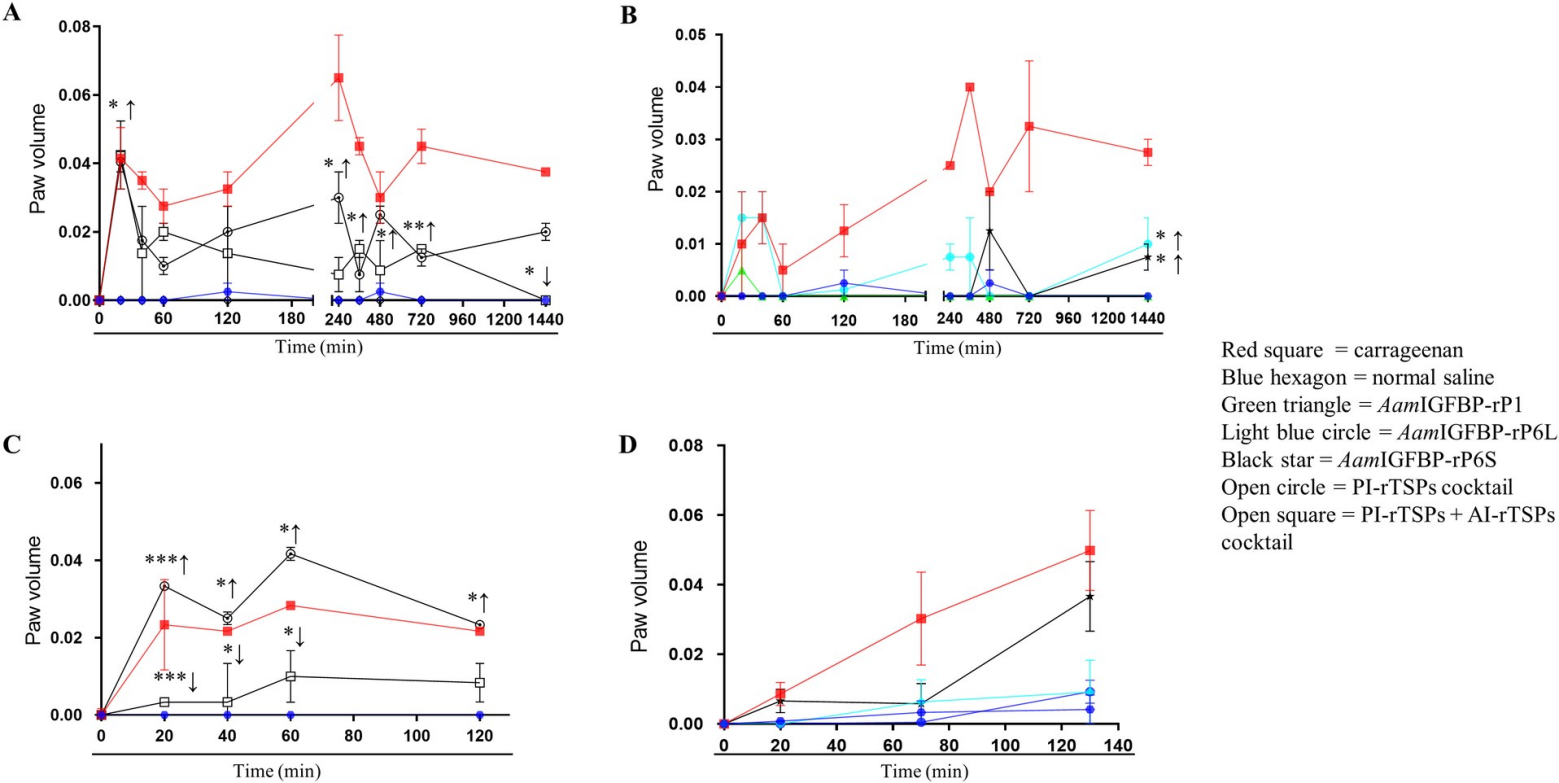
Cell culture validated PI-rTSP (r*Aam*IGFBP-rP1, r*Aam*IGFBP-rP6S, and r*Aam*IGFBP-rPL6) and AI-rTSP (rAAS27 and rAAS41) are functional *in vivo*. Line diagram showing increase in paw swelling in mice injected with high dose (25 μg: A, B) and low dose (10 μg: C, D) of cocktail or individual r*Aam*IGFBP-rP1, r*Aam*IGFBP-rP6L, and r*Aam*IGFBP-rP6S or rAAS27 and rAAS41. Data is represented as mean of three biological replicates ± SEM. Filled red squares = carrageenan injected, blue hexagon = normal saline, green filled hexagon, triangle = r*Aam*IGBP-rP1 injected groups, light blue circle = r*Aam*IGBP-rP6L, Black star = r*Aam*IGBP-rP6S, Open circle = PI-rTSP cocktail, Open square = PI-rTSPs + AI-rTSPs cocktail (*) = p ≤ 0.05, (**) = p ≤ 0.01, (***) = p ≤ 0.001, (****) = p ≤ 0.0001 indicating statistically significant difference between normal saline injected group and treatments groups. No asterisks indicated represent non-statistical significance. Arrow (↑) indicate increase in paw volumes compared to normal saline control and arrow (↓) indicate decrease in paw volumes compared to PI-rTSPs injected paws.

Paw edema induction confirmed that cell culture validated PI-rTSP and AI-rTSP were functional *in vivo* (Fig 8). The high dose (25 μg) of the PI-rTSP cocktail progressively induced edema through 24 h (Fig 8A). Four statistically significant edema points were observed at 20 min (p≤0.05), 240 min (p≤0.05), 480 min (p≤0.05), and at 1440 min (p≤0.01) in PI-rTSPs and AI-rTSPs treatments. In contrast, co-injecting high dose of AI-rTSP cocktail (25 μg) significantly suppressed PI-rTSP-induced edema at 120 (p≤0.05), 240, 480, and 1440 mins, respectively. Injection of individual r*Aam*IGFBP-rP6L (25 μg) caused edema that peaked at 480 min and 1440 min (p≤0.05), while r*Aam*IGFBP-rP6S induced edema that peaked at 20 min, 40 min, 240 min, and 1440 min (p≤0.05)] (Fig 8B). On the other hand, r*Aam*IGFBP-rP1 did not cause detectable edema (Fig 8B).

To rule out the effect of too much protein being injected, we repeated the assay with 10 μg of the low dose cocktail. Consistent with the high dose, injection of 10 μg of the PI-rTSP cocktail induced significant edema that was observed at 20 (p≤0.001) min and at 60 min (p≤0.05) (Fig 8C). Likewise, when co-administered with low dose (10 μg) AI-rTSP cocktail, edema was significantly reduced at 20 (p≤0.001), 40 (p≤0.05), 60 (p≤0.05) and 120 min (Fig 8C). In animals that were injected with individual PI-rTSPs (10 μg), r*Aam*IGFBP-rP6S induced edema that was observed between 20–130 min, while r*Aam*IGFBP-rP6L induced edema that was observed at 130 min (Fig 8D), but not r*Aam*IGFBP-rP1.

### PI-rTSP and AI-rTSP differentially regulated inflammatory markers in mice paws

**Fig 9:**
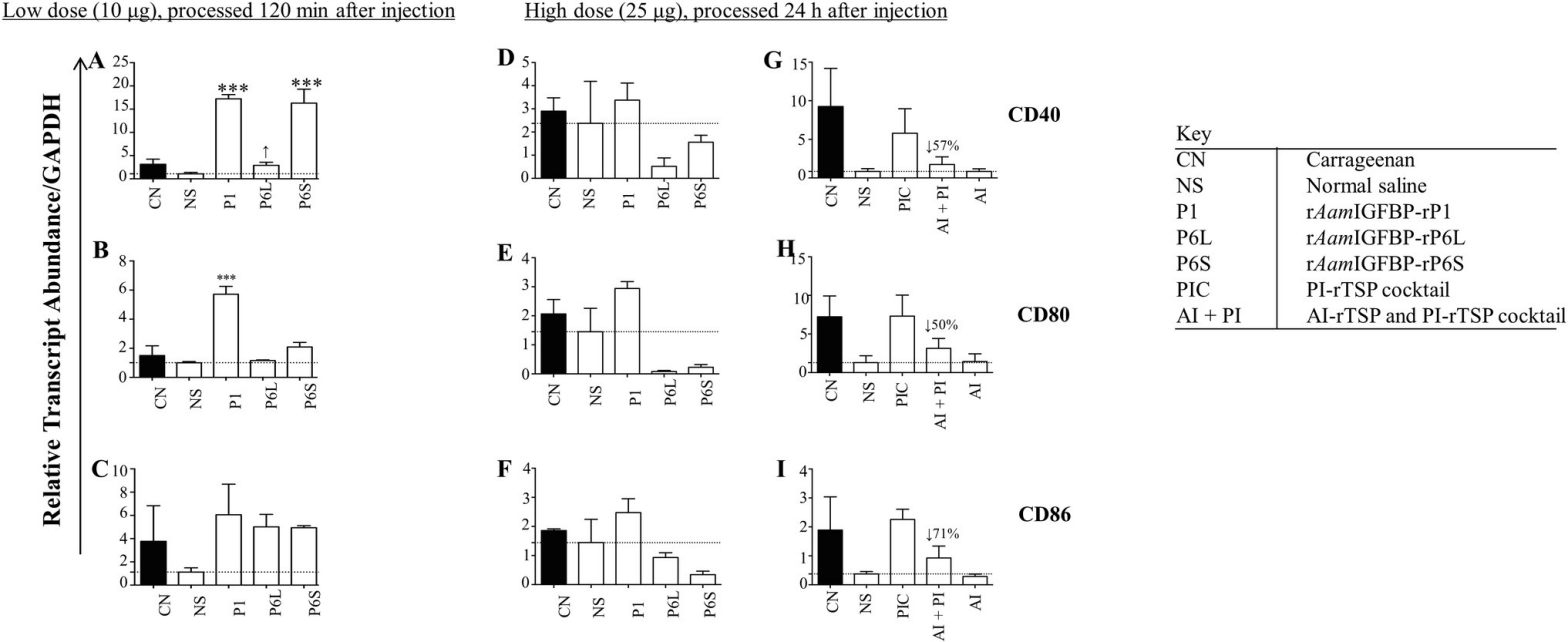
*A*. *americanum* tick PI-rTSP induce expression of co-stimulatory markers *in vivo*, AI-rTSPs suppress. Relative transcript abundance of PI co-stimulatory markers, CD40, CD80 and CD86 in low dose (A, B, and C) and high dose (D, E, F, G, H, I) injected paws. Using Glyceraldehyde-3-phosphate (GAPDH) as the reference gene, relative transcript abundance was determined using comparative Ct (ΔΔ Ct) method. Data is reported as Mean (M) of two biological replicates ± SEM. Asterisks (*) = p ≤ 0.05, (**) = p ≤ 0.01, (***) = p ≤ 0.001, (****) = p ≤ 0.0001 indicating statistically significant difference between normal saline injected group and treatments groups. No asterisks indicated represent non-statistical significance. Arrow (↓) indicate decrease in cytokine transcript abundance compared to normal saline controls.

**Fig 10:**
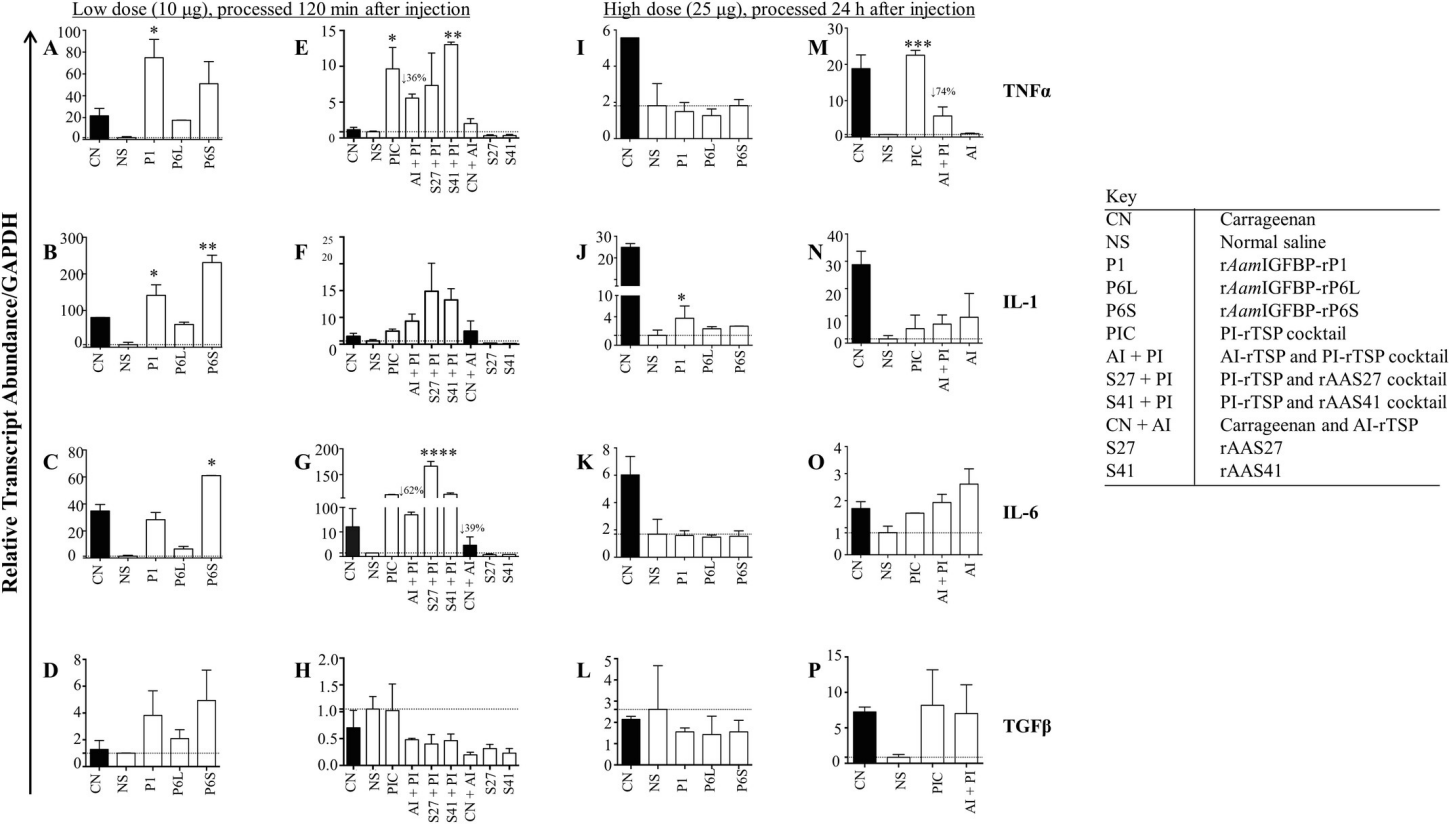
*A*. *americanum* tick PI-rTSP induce expression of pro-inflammation cytokines *in vivo*, AI-rTSPs suppresses. Relative transcript abundance of cytokines in paws that were treated with low dose individual PI-rTSPs (A, B, C, and D) or with cocktail PI-rTSP or Carageenan followed by individual or cocktail AI-rTSP (E, F, G, and H). Fig 10I, 10J, 10K and 10L = paws that were injected with high dose individual PI-rTSPs; and Fig 10M, 10N, 10O and 10P = were injected with high dose PI-rTSPs and AI-rTSP cocktail. Data is reported as Mean (M) of two biological replicates ± SEM. Asterisks (*) = p ≤ 0.05, (**) = p ≤ 0.01, (***) = p ≤ 0.001, (****) = p ≤ 0.0001 indicating statistically significant difference between normal saline injected group and treatments groups. No asterisks mean non-significance. Arrow (↓) indicate decrease in cytokine transcript abundance compared to normal saline controls.

**Fig 11:**
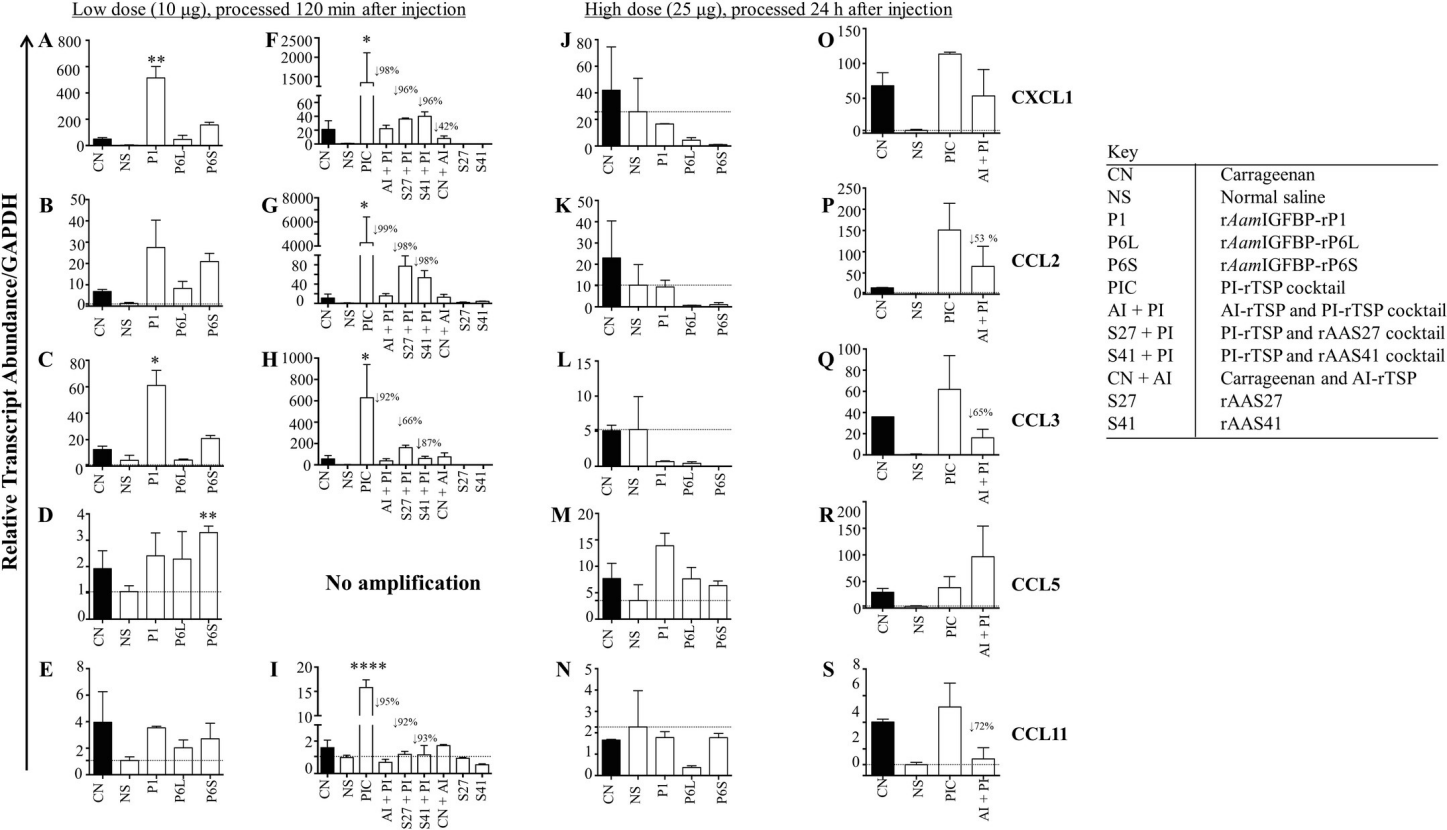
*A*. *americanum* tick PI-rTSP induce expression of chemokines *in vivo*, AI-rTSPs suppresses. Relative transcript abundance of chemokines in paws that were injected with low dose individual (A, B, C, D and E) or with cocktail PI-rTSP or Carrageenan followed by individual or cocktail AI-rTSP (F, G, H, I). Fig 11J, 11K, 11L, 11M and 11N = paws that were injected with high dose individual PI-rTSPs; and Fig 11O, 11P, 11Q, 11R and 11S = paws that were injected with high dose PI-rTSPs and AI-rTSP cocktail. Data is reported as Mean (M) of two biological replicates ± SEM. Asterisks (*) = p ≤ 0.05, (**) = p ≤ 0.01, (***) = p ≤ 0.001, (****) = p ≤ 0.0001 indicating statistically significant difference between normal saline injected group and treatments groups. No asterisks indicated represent non-statistical significance. Arrow (↓) indicate decrease in chemokine transcript abundance compared to normal saline controls.

Effects of PI-rTSPs and AI-rTSPs on inflammation marker expression were investigated by qPCR (Figs 9–11). Consistent with cell culture data (Fig 2), PI co-stimulatory markers, CD40, CD80, and CD86 were up regulated in mice that were injected with low dose individual PI-rTSPs (10 μg) and processed at 120 min post injection (Fig 9A, 9B and 9C). Expression of CD40 was significantly enhanced in mice that were injected with low dose r*Aam*IGFBP-rP1 and r*Aam*IGFBP-rP6L, but not r*Aam*IGFBP-rP6S (Fig 9A). Likewise, CD80 was significantly induced by low dose r*Aam*IGFBP-rP1 but not r*Aam*IGFBP-r6L and r*Aam*IGFBP-rP6S (Fig 9B). For CD86, induction was apparent but it was not significant (Fig 9C). In mice that were injected with high dose individual PI-rTSP (25 μg) and processed at 24 h post injection, all three co-stimulatory markers were induced (but not significantly) in r*Aam*IGFBP-rP1-injected animals, but not r*Aam*IGFBP-r6L and r*Aam*IGFBP-rP6S (Fig 9D, 9E and 9F). In contrast, injecting high dose of the PI-rTSP cocktail significantly induced expression of CD40, CD80, and CD86 above normal saline control, while co-injecting with the high dose of AI rTSP cocktail suppressed transcription of CD40 and CD86 by 70% and by 50% for CD80 (Fig 9G, 9H and 9I).

Fig 10 summarizes expression of PI and AI cytokines in PI-rTSP induced edema. TNF-α, IL-1, and IL-6 were significantly expressed in low dose individual (Fig 10A, 10B and 10C) and PI-rTSP cocktail (Fig 10E, 10F and 10G) treatment. In contrast, except for IL-1, which was slightly enhanced (Fig 10J), expression of TNF-α and IL-6 was below negative control in animals that were injected with high dose (25 μg) of the individual PI-rTSP (Fig 10I and 10K). Except for TNF-α which was significantly up regulated above normal saline (Fig 10M), other transcripts were apparently up regulated but not significantly (Fig 10N, 10O and 10P).

Co-injecting with the low dose of AI-rTSP had mixed effects on expression of the four cytokines (Fig 10E–10H). In low dose-treated mice, co-injecting AI-rTSP suppressed TNFα by ~35% and IL-6 by 62% (Fig 10E and 10G), but not IL-1 (Fig 10F). Likewise, co-injecting with high dose AI-rTSP reduced expression of TNFα by 74% (Fig 10M), while IL-1 and IL-6 were apparently enhanced but not significantly (Fig 10N and 10O). Also notable, the TGFβ transcript was induced in animals that were injected with low dose of the individual PI-rTSPs (Fig 10D) as well as the high dose of the PI-rTSP cocktail (Fig 10P), but not the low dose cocktail (Fig 10H) and high dose individual PI-rTSP (Fig 10L).

The effects of PI-rTSPs and AI-rTSPs on chemokine transcription mirrored cytokine expression. Notably, chemokine transcription was significantly upregulated in mice that were injected with low dose, individual (11A, B, C, D, E) or cocktail (Fig 11F, 11G, 11H and 11I) PI-rTSP with exception of CCL5, for which there was no amplification. Except for CCL5 (Fig 11M), which was upregulated in high dose of the individual PI-rTSPs, other chemokines tested were expressed below normal saline injected controls (Fig 11J, 11K, 11L and 11N). In animals that were injected with high dose of the PI-rTSP cocktail, all chemokines were up regulated (Fig 11O, 11P, 11Q, 11R and 11S). Also, notable, co-injecting with the high dose AI-rTSP cocktail suppressed expression of CXCL1, CCL2, CCL3, and CCL11 by ~50%, 53%, 65%, and 72% respectively (Fig 11O, 11P, 11Q and 11S) and CCL5 which was enhanced (Fig 11R).

## Discussion

Hard ticks successfully feed and transmit tick borne disease (TBD) agents by secreting numerous tick saliva proteins (TSPs) to thwart the host’s immune defenses, which would otherwise reject the tick. Thus, discovery of tick immuno-modulatory TSPs is highly sought after as these might serve as targets in tick-antigen based vaccines to prevent TBD infections. This study provides evidence to suggest that *A*. *americanum* ticks might utilize countervailing functions of PI proteins to regulate the evasion of host immune defenses. Our *in vitro* and *in vivo* data in this study has demonstrate that *Aam*IGFBP-rP-1, *Aam*IGFBP-rP6S, and *Aam*IGFBP-rP6L (38, 39) activated MΦ to express PI markers, whereas *A*. *americanum* AAS27 and AAS41 suppressed expression of PI markers by activated MΦ. This study builds on our previous studies that showed that *Aam*IGFBP-rP-1, *Aam*IGFBP-rP6S, and *Aam*IGFBP-rP6L were up regulated when ticks were stimulated to start feeding [38, 41], were immunogenic and injected into the host within 24–48 h after attachment [42], and if disrupted by RNAi silencing prevented successful tick feeding [43]. Likewise, the observed AI effects of rAAS27 and rAAS41 in this study is in agreement with our recent studies that showed that AAS27 might inhibit inflammation by targeting trypsin and plasmin and AAS41 by targeting chymase and chymotrypsin [44].

Although not yet determined at the tick feeding site, activated MΦ at an inflamed site occur in a spectrum that is bordered by classically activated PI (M1) and alternately activated (M2) anti-inflammation phenotypes [45]. Broadly, the M1 phenotype is induced at the front end of an immune reaction, and the M2 at the tail end of an immune response to resolve the inflammatory response. M1 MΦ express multiple factors that drive inflammation including co-stimulatory markers, CD40, CD80, and CD86, cytokines such as TNF-α, IL-1β, IL-6, and IL-12, oxygen intermediates and reactive nitrogen species such as nitric oxide (NO) [46–50]. On the other hand, M2 MΦ express factors that lead to resolution of inflammation to protect against self-injury (50). On this basis, we speculate that PI-rTSPs in this study might activate skin resident MΦ to the M1 phenotype to drive local inflammation, while AI-rTSPs, which enhanced expression of anti-inflammation markers might play roles in resolution or moderation of TSP-induced inflammation. The observation that both rAAS27 and rAAS41 reduced the expression of PI markers by both LPS- and PI-rTSPs-activated MΦ was not surprising in that several studies have previously reported tick immuno-suppressive effects against skin immune cells in tick saliva [51–55]. However, the findings that rAAS27 and rAAS41 did not apparently affect non-activated MΦ, but selectively affected functions of MΦ that were spontaneously activated or pre-activated by LPS and PI-rTSP was intriguing. From the perspective of tick feeding physiology and tick transmission of TBD agents, data in this study raise interesting future research questions. For instance, it would be interesting to further explore the effect of rAAS27 and rAAS41 reversing MΦ that were pre-activated by PI-rTSP on the outcome of an immune response to TBD agents. A notable observation in our flow cytometry data was that, AI-rTSPs were much more effective against LPS-activated MΦ than PI-rTSP. Interestingly, TBD agents such as *B*. *afzelii*, *B*. *burgdorferi* [56, 57], *E*. *chaffeensis* [58], *A*. *phagocytophilum* [59] and, *Powassan virus* [60] can activate MΦ to express high levels of PI cytokines. However, this does not seem to limit colonization of the host by transmitted TBD agents. Also, interestingly, clinical outcomes of TBD infections are more pronounced when TBD agents are co-inoculated with tick saliva proteins [31]. Given the finding that rAAS27 and rAAS41 reversed activated MΦ, it is possible that AI-TSPs described here could prevent MΦ from killing transmitted TBD agents, which ultimately could aid pathogen transmission. Several studies have reported that tick saliva/SGE of different tick species including *A*. *variegatum*, *I*. *scapularis*, *R*. *microplus*, *R*. *appendiculatus*, *R*. *sanguineous*, and *D*. *variabilis* suppressed expression of PI markers by MΦ that were first activated by LPS or were exposed to pathogens [29, 61–64]. Given our observations that AAS27 and AAS41 suppressed expression of pro-inflammation markers by LPS-activated MΦ, it is potentially possible that the immunosuppressive effects observed in tick saliva/SGE of different tick species could be mediated by AAS27 and AAS41 homologs.

Skin inflammation in response to tick bites in humans and rodents were characterized by high expression of multiple PI cytokines and chemokines [65–67]. On this basis, we suspect that high expression of PI cytokines and chemokines in PI-rTSP-induced edema suggests that these proteins could play roles in mediating tick and host interactions. An inflammatory response is characterized by infiltration of multiple innate immune cells including mast cells, MΦ, and neutrophils [41, 68–70]. Although we did not conduct immuno-histochemical staining and cellular imaging of inflamed paws to enumerate immune cells, the observed high expression of cytokines and chemokines, which are expressed by various immune cells possibly suggest an influx of immune cells into PI-rTSP injected paws. Specifically, the observation of high expression of PI co-stimulatory markers, CD40, CD80, and CD86, which are mostly produced by the M1 MΦ phenotype [71], could suggest the presence of these cells in injected paws. Chemokines that were investigated in this study were selected for their roles in attracting immune cells to the site of inflammation [72, 73]. Therefore, the observed high expression of chemokines suggested that the edema formation was a result of various cellular migration at the inflamed site.

The findings in this study are not unique as pro-inflammation tick proteins including a tick histamine release factor and an 84 kDa tick serine protease, were previously reported [74, 75]. Given the expectation that in order to complete feeding, ticks must evade the host’s innate immune defense response against tick feeding that also include inflammation, our findings in this study may be considered counterintuitive. However, from the perspective of tick feeding physiology, inflammation can be beneficial to tick feeding as it might lead to increased blood flow into the feeding site benefiting the tick in acquiring the blood meal. Additionally, the increased flow of inflammatory cell monocytes into the inflamed tick feeding site might result in enhanced transmission of *A*. *americanum* transmitted TBD agents such as Bourbon virus, Heartland virus, and *E*. *chaffeensis* that have tropisms for inflammatory cells including MΦ [76, 77].

It is also important to note that treating MΦ with high dose (10 μg) of PI-rTSP cocktail also induced expression of an anti-inflammation cytokines, IL-10, which conforms to the M2 MΦ phenotype. We are of the view that, the higher concentration of PI-rTSP over-stimulated MΦ to the extent the correct anti-inflammation response was triggered. Similarly, we also observed that, with exception of TNFα, other cytokines, IL-1, IL-6, IL-10, and TGFβ were enhanced in MΦ that were first treated with PI-rTSP for 24 h followed by AI-rTSP for another 24 h. Whether or not, these findings occur physiologically remains to be investigated. Like most functional analysis studies of recombinant tick saliva proteins, the limitation is that the amount of native TSPs that are injected into the host during tick feeding is unknown, and thus future studies to define functional roles of native proteins are required.

In conclusion, we have proposed that *A*. *americanum* tick might utilize countervailing functions of pro- and anti- inflammatory proteins to regulate evasion of host defenses. Our proposal is that the tick first secrets PI-TSPs that stimulate MΦ into the pro-host defense phenotype and then secretes AI-TSPs to de-activate the activated immune cells including MΦ. This proposal assumes that proteins in this study were sequentially secreted during tick feeding. The findings in this study warrants further investigations into functional roles of proteins in this study in transmission of TBD agents, and their utility as anti-tick vaccine antigens.

## Materials and methods

### Ethical statement

Healthy, pathogen-free BALB/c mice were purchased from Charles River laboratories (Wilmington, MA). Animal experiments were done according to the animal use protocol (#2015–0079) approved by Texas A&M University Institutional Animal Care and Use Committee (IACUC) that meets all federal requirements in Animal Welfare Act (AWA), the Public Health Service Policy (PHS), and the Humane Care and use of laboratory animals.

### Expression of *A*. *americanum* tick saliva insulin-like growth factor binding proteins-related proteins (*Aam*IGFBP-rP) in Human Embryonic Kidney (HEK)

Preliminary assays revealed that insect-cell expressed recombinant (r) *Aam*IGFBP-rP1,—*Aam*IGFBP-rP6L, and *Aam*IGFBP-rP6S (with endotoxins removed) activated both immortalized and PBMC derived macrophages (MΦ) to express PI markers. To rule out the possibility of endotoxin involvement in the observed results, r*Aam*IGFBP-rP1, r*Aam*IGFBP-rP6L, r*Aam*IGFBP-rP6S rTSPs were re-expressed in Human Embryonic Kidney (HEK 293) mammalian cells using the pcDNA 3.3 expression plasmid (Thermo-Scientific). Modified pcDNA 3.3 with added CD5 secretion signal to allow secretion of the recombinant protein into spent media was kindly provided by Dr. Mwangi (Kansas State University, Kansas, USA). Mature protein encoding open reading frames was sub-cloned into the modified pcDNA3.3 plasmid using primers listed in Table 1. The reverse primer included the flag tag sequence (DYKDDDDK) which was used for detection and affinity purification of the recombinant protein. Recombinant plasmids were transformed into *E*.*coli* DH5α cells and subsequently purified using plasmid miniprep kit (Omega) followed by quantification and transfection. HEK-293A adherent cells were used in pilot expression, and the HEK-293 Freestyle (HEK-293F) cell line (Thermo-Scientific) was used for large-scale rTSP production in suspension cultures [78].

**Table 1:** Expression primers of *Amblyomma americanum* tick saliva insulin-like growth factor binding proteins-related proteins in HEK cells.

*Amblyomma americanum* rTSP	PRIMER SEQUENCE (Restriction sites underlined, FLAG Tag and linker sequence highlighted in grey)
r*Aam*IGFBP-rP1	For:5′GGATCCTCGCAAGGAGTGCGGGCCTTG 3′ Rev:5′GGATCCCTACTTATCGTCATCGTCCTTGTAGTCTTTTT TGGGCAGCACGTTGAGCTTGG 3′
r*Aam*IGFBP-rP6S	For:5′GGATCCTACGTCGGAACCGCACTGCG 3′ Rev:5′GGATCCCTACTTATCGTCATCGTCCTTGTAGTCTTTTTTCTCGTGATGGGCCGAGTCG 3′
r*Aam*IGFBP-rP6L	For: 5′GGATCCTACGTCGGAACCGCACTGCGAGG 3′ Rev:5′GGATCCCTACTTATCGTCATCGTCCTTGTAGTCCTCGTGGTGGGCCGAGTCGCCGCCG 3′

Adherent 293A cells were grown to a monolayer of up to ~60–70% confluence in T-75 flasks containing 10 mL of Dulbecco’s modified Eagle’s medium (DMEM) (Lonza) supplemented with glutamine and heat-inactivated fetal bovine serum (10%) at 37°C with 5% carbon dioxide (CO_2_) and 85–90% humidity. For transfection, 1 μg of plasmid DNA and 2.4 μL of the transfection reagent, polyethyleneamine (PEI) diluted in 180 μL of Opti-MEM (Thermo-Scientific) medium was incubated for 20 min at room temperature (RT). Following the incubation, 180 μL of the plasmid DNA-PEI mixture was added to each plate containing the cells, mixed gently by rocking the plate, and incubated for ~48 h at 37°C in the humidified incubator with 5% CO_2_ [79].

Expression was validated by immuno-staining of HEK cells using the monoclonal antibody to FLAG (5 μg/mL) (Sigma-Aldrich). Fast red was used to (4-Chloro-2-methylbenzenediazonium salt, Naphthol, Sigma-Aldrich) stain the cells, which were visualized using inverted microscope. Spent media was subjected to routine ELISA using the HRP (Horse-radish peroxidase) conjugated antibody to FLAG-Tag (Sigma-Aldrich) to verify secretion of the recombinant product into culture media.

For large-scale expression, HEK-293F suspension cultures were grown to mid-logarithmic phase with shaking at 125 rpm in 8% CO_2_ and 85% relative humidity. The cells were then seeded at 30 × 10^6^ viable cells in 293 Freestyle media (Thermo-Scientific), allowed to incubate for 2 h. Recombinant plasmids (30 μg each) were incubated in 1 mL Opti-MEM media (Thermo-Scientific) separately. Following incubation, 293fectin transfection reagent (Thermo-Scientific) and recombinant plasmids were combined and incubated for an additional 30 min and then added to 30 mL cell culture containing 30 million HEK-293F cells. The cell suspension was harvested after 72 h and recombinant proteins were purified using FLAG M2 affinity gel purification (Sigma). Bound recombinant proteins were eluted in 1 mL fractions under acidic conditions using 0.1M Glycine buffer (pH 3.5) and immediately neutralized with 25 μL 1M Tris-HCl (pH 8.0). Purification was confirmed using routine SDS-PAGE and Coomassie blue or silver staining. Subsequently, relevant fractions were combined, concentrated and dialyzed against Tris-NaCl buffer (50 mM Tris, 150mM NaCl, pH 7.4) using the 10 kDa molecular cut off membrane spin filters (Pall Life Sciences). Expression recombinant AAS27 and AAS41 is reported elsewhere [44].

### Macrophage cell activation assays

This was done using bovine PBMC differentiated and immortalized RAW 267 MΦ (ATCC). In the preliminary assay, PBMCs were isolated from bovine peripheral blood using Ficoll-Paque solution (Sigma-Aldrich). Monocytes from the PBMCs were isolated by Magnetic sorting method using primary mouse anti-bovine CD14 (Washington State University) antibody and a secondary antibody, anti-mouse IgG microbeads (Miltenyi Biotec). The cells were labelled with the anti-CD14 antibody and then loaded onto a MACS column, for CD14 labelled monocytes. The monocytes were counted and allowed to differentiate into macrophages for 6–8 days. Cell morphology was visually confirmed using an inverted microscope (Nikon). Following differentiation, the assay was set up using individual concentrations of 10 μg /mL, 1 μg/mL and 0.1 μg/mL of insect cell expressed r*Aam*IGFBP-rP1, r*Aam*IGFBP-rP6L, r*Aam*IGFBP-rP6S [39] and yeast expressed *A*. *americanum* serpin (AAS) 27 and 41 (44). Following 24 h, the cells were collected and stained for CD40, CD80, and CD86 expression and then analyzed by flow cytometry (described below). Lipopolysaccharide (LPS), a bacterial cell wall component was used as positive controls and media alone served as a negative control. All assays were performed in triplicates for each time.

Subsequent to the preliminary assay, RAW 264.7 MΦ (ATCC) were used in the assay. Routinely, cells were grown in Dulbecco’s Modified Eagle’s Medium (DMEM) (Lonza) supplemented with 4 mM L-glutamine, 4500 mg/L glucose, 1 mM sodium pyruvate, sodium bicarbonate, non-essential amino acid solution (Thermo-Scientific) and 10% fetal bovine serum (FBS) (Thermo-Scientific). Routinely, MΦ were seeded in 12 or 24 well plates overnight and cultured at 37°C with 5% CO_2_, 85–90% humidity overnight to approximately 80% confluency. For pro-inflammation activation assays, 10^6^ MΦ were incubated with 0.10, 1.0, and 10 μg/mL of affinity purified HEK cell expressed r*Aam*IGFBP-rP1, r*Aam*IGFBP-rP6L, and r*Aam*IGFBP-rP6S.

In preliminary analysis, we had observed that pro-inflammation markers were expressed at below background in MΦ that were treated with various dosages of rAAS27 and rAAS41. To investigate this, we conducted two assays. In the first assay, expression of pro-inflammation makers was determined in MΦ that were co-cultured with various dosages (0.1, 1.0, 10 μg/mL of the mixture of rAAS27 or rAAS41 and LPS or r*Am*IGFPB-rPs. In the second assay, MΦ were first activated with LPS (100 ng) or various dosages (0.1, 1.0, 10 μg/mL) of r*Aam*IGFBP-rP1, r*Aam*IGFBP-rP6L and r*Aam*IGFBP-rP6S for 24 h before replacing spent culture media with fresh media supplemented with various dosages (0.1, 1.0, 10 μg/mL) of individual or cocktail mix of rAAS27 and rAAS41. At 24 and 48 h post incubation, spent cultures were processed for assays described below.

### Cell surface marker staining and flow cytometry

Treated and non-treated MΦ, were detached, and re-suspended in staining medium (DMEM with sodium azide). Immuno-labeling of bovine (preliminary analysis) and murine cell surface markers was performed by incubating cells with 15 μg/mL fluorescein isothiocyanate (FITC) conjugated antibodies to CD40 (Abcam), CD86 and CD80 (Thermo-Scientific) and isotype matched control mAbs IgG2a and IgG2b (Abcam) for 30 min on ice. After incubation, cells were washed three times with DMEM media containing 0.01% sodium azide and re-suspended in 400 μL 1X PBS (pH-7.4) containing 1 μg/mL propidium iodide, for excluding dead cells, and analyzed by flow cytometry with parameters set to 10,000 events, filter setting 530/30 nm wavelength (FACS Caliber) using acquisition software BD CellQuest (BD Biosciences) and the analysis program FlowJo 9.8.5 (TreeStar) at digital imaging core facility (College of Veterinary Medicine, Texas A&M University, College Station, TX).

### Nitric oxide and Cytokine detection in the spent media

Nitric oxide metabolites released in the cell culture supernatant as nitrate or nitrite was detected by Total Nitric Oxide detection kit (Thermo-Scientific) according to the manufacturer’s protocol. Photometric measurement of the absorbance due to this azo chromophore determined the NO_2_^-^ (nitrite) concentration at 540 nm wavelength using an ELISA plate reader (BioTek Instruments).

Cytokine ELISA of TNFα, IL-1, IL-6, IL-10, IL-12, and TGFβ was done using specific antibodies (Ready-SET-Go!, eBioscience, Thermo-Scientific). Optical densities were measured using an ELISA plate reader at 450 nm wavelength (BioTek Instruments, Inc).

### MΦ phenotype staining

RAW 264.7 MΦ were seeded on Nunc Lab-Tek Chamber Slide (Thermo-Scientific) in the presence of the cocktail (10 μg/mL) of r*Aam*IGFBP-rP1, r*Aam*IGFBP-rP6L, and r*Aam*IGFBP-rP6S, or positive control (100 ng LPS), or negative control (media with buffer) for 24 h. The cells were then fixed with ice cold methanol, blocked with 1% BSA, 22.52 mg/ml glycine in PBST (PBS+0.1% Tween 20) for 30 min and immuno-stained with the antibody to Actin (Abcam). The following day, the cells were washed in 1 X PBS and incubated with secondary antibody conjugated with Alexa Fluor 488 (Abcam) for 1 h in the dark. The cells were washed, treated with mounting media containing DAPI (Thermo-Scientific) for nuclei staining and cell staining was analyzed by confocal microscopy. Images were acquired with Ziess LSM 780 confocal microscope and merged using Zen 2012 SP1 (black edition) software in the Imaging facility (College of Veterinary Medicine, Texas A&M University, College Station, TX).

### Paw edema assay

Retired female breeder BALB/c mice (Envigo) were maintained for one week to acclimatize prior to experiment. The carrageenan-induced hind paw inflammation model was used to investigate the PI and AI role of rTSPs [80]. Prior to each injection, the basal footpad volume was recorded using a plythesmometer (Harvard Apparatus). Four experiments were conducted using three mice per group. In the first experiment, effects of a high dose cocktail of r*Aam*IGFBP-rP1, r*Aam*IGFBP-rP6L, and r*Aam*IGFBP-rP6S was assessed. To make the high dose cocktail, approximately 40 μg each of endotoxin free mammalian cells expressed r*Aam*IGFBP-rP1, r*Aam*IGFBP-rP6L and r*Aam*IGFBP-rP6S were combined, and concentrated to reduce volume using Jumbosep centrifugal spin filter devices (Pall Life Sciences). Similarly, 40 μg each of endotoxin free rAAS27 and rAAS41 were combined and concentrated as above. The first group of mice received 25 μg of the r*Aam*IGFBP-rP1, r*Aam*IGFBP-rP6S and r*Aam*IGFBP-rP6L cocktail (PI-rTSP group). The second group of mice received 25 μg cocktail of rAAS27 and rAAS41 cocktail (AI-rTSP group), third group received 25 μg of the AI-rTSP and PI-rTSP cocktail. In the second experiment, the effects of high dose (40 μg per mouse) of r*Aam*IGFBP-rP1, r*Aam*IGFBP-rP6L and r*Aam*IGFBP-rP6S individually were assessed.

In the third and fourth experiments, mice were injected with low doses of cocktail of individual proteins. To make the low dose cocktail, 10 μg each of r*Aam*IGFBP-rP1, r*Aam*IGFBP-rP6L and r*Aam*IGFBP-rP6S with or without 10 μg of rAAS27 and rAAS41 cocktail were prepared. In the third experiment, the first group of mice received the 10 μg the PI-rTSP cocktail, the second received the 10 μg of the AI-rTSP cocktail, the third group received 10 μg of AI-rTSP and PI-rTSP cocktail, the fourth group received 10 μg of rAAS27 or rAAS46 individually. In the fourth experiment, each group of mice received 10 μg of *Aam*IGFBP-rP1, *Aam*IGFBP-rP6L and *Aam*IGFBP-rP6S. For all experiments, algae derived inflammation agonist, carrageenan (2% w/v in 0.9% saline) was used as positive control and normal saline (9 g/L NaCl) or 150mM 50mM Tris NaCl buffer (pH 7.4) as negative control (Table 2).

**Table 2:** Treatment groups for Paw edema assay.

Experiment (min)	Treatment groups	Dose injected	Diluent: Normal Saline (NS) or 150mM NaCl 50 mM Tris pH 7.4 (TB)
1 –(1440 min)	Carrageenan (C)	2% (weight/volume)	NS
	Normal Saline (NS)		NS
	PI-rTSPs	25 μg	TB
	PI-AI rTSPs	25 μg PI + 25 μg AI rTSPs	TB
2 –(1440 min)	C	2%	NS
	S	0.9%	NS
	r*Aam* IGFBP-rP1	25 μg	TB
	r*Aam* IGFBP-rP6 L	25 μg	TB
	r*Aam* IGFBP-rP6 S	25 μg	TB
3 –(120 min)	C	2% (w/v)	TB
	NS	0.9% (w/v)	TB
	PI-rTSPs	10 μg	TB
	PI-AI-rTSPs	10 μg PI-rTSPs + 10 μg AI-rTSPs	TB
	PI-AAS27	10 μg PI-rTSPs + 10 μg AAS27	TB
	PI-AAS41	10 μg PI-rTSPs + 10 μg AAS41	TB
	C + AI-rTSPs	2% C (w/v) + 10 μg AI-rTSPs	TB
	AAS27	10 μg	TB
	AAS41	10 μg	TB
4 –(130 min)	C	2% (w/v)	TB
	NS	0.9% (w/v)	TB
	*Aam* IGFBP-rP1	10 μg	TB
	*Aam* IGFBP-rP6 L	10 μg	TB
	*Aam* IGFBP-rP6 S	10 μg	TB

As index of edema formation, the first and second experiment inflammation was measured at times 0 (before injection), 20, 40, 60, 120, 240, 360, 720, and 1440 min’ post injection. For the third and fourth experiments, paw edema was measured at 0, (before injection), 20, 40, 60, and 120 or 130 min’ post injection. After measurements, the mice were euthanized (3L/minute, CO_2_) and injected and non-injected paws were placed in cryotubes and snap frozen in liquid nitrogen. The paws were collected at the level of calcaneus bone for cytokine, chemokine, and myeloperoxidase assays.

### Quantitative RT-PCR

Total RNA was routinely extracted using the TRIzol reagent according to instructions (Thermo-Scientific). From MΦ, cells were lysed directly into 1 mL TRIzol. For paws, tissues were minced in 1 mL TRIzol solution using sterile soft tissue scissors followed by sonication using the tissue dismembranator (VWR). Total RNA from paws were subjected to mRNA isolation using OligodT magnetic beads (Thermo-Scientific) and bound mRNA was eluted using elution buffer (10mM Tris-HCl, pH 7.5). The NanoDrop (BioTek Instruments, Inc) was used to determine quantity and quality of total RNA for cDNA synthesis.

Template cDNA was synthesized from 500 ng total RNA and 25 ng mRNA using the Verso cDNA synthesis kit (Thermo-Scientific). The Verso cDNA synthesis kit contains an enhancer reagent, which prevents genomic (g) DNA carryover into the synthesized cDNA. The reverse transcription cDNA synthesis step included incubation at 42°C from 30 min and 95°C for 2 min in the thermocycler (Bio-Rad). After cDNA synthesis, the working stock for each sample was diluted 1:10 with DEPC treated water.

The qPCR (Quantitative polymerase chain reaction) was performed in triplicates in a 50 μl final reaction mix containing 3 μl each specific primers (300 nM each, Table 3), 5 μl 1:10 diluted template cDNA, and 25 μl 2X SYBR (Thermo-Scientific) green PCR master mix [(Applied Biosystems 7300 Real Time PCR System (Thermo-Scientific) and Bio-Rad qPCR machine (Bio-Rad)]. Settings were: 50°C for 2 min for one cycle followed by 95°C for 10 min, 95°C for 40 cycles at 15 seconds’ interval and 60^º^C for 1 minute. Cognate mRNA expression levels were determined using the comparative delta Δ C_t_ method [81]. The GAPDH (Glyceraldehyde 3-phosphate dehydrogenase) gene was used as an internal reference gene. Amplifications from non-treated controls were used as calibrator for *in vitro* experiments and normal saline injected controls for *in vivo* experiments. For each biological replicate, we did qPCR in three technical triplicates.

**Table 3:** Primers for quantitative RT-PCR of pro and anti-inflammatory cytokines, co-stimulatory markers and chemokine expression.

Immune molecule	Target gene	Primer sequence	References
**Pro-inflammatory cytokines**	**TNF-α**	For: 5′ATGAGCACAGAAAGCATGA 3′ Rev: 5′GAATGAGAAGAGGCTGAGA 3′	[82]
	**IL-6**	For: 5′CTCTGGGAAATCGTGGAAAT 3′ Rev: 5′CCAGTTTGGTAGCATCCATC 3′	[83]
	**IL-1**	For:5′CAACCAACAAGTGATATTCTCCATG 3′ Rev: 5′GATCCACACTCTCCAGCTGCA 3′	[84]
**Anti-inflammatory cytokine**	**IL-10**	For: 5′GGGAAGACAATAACTGCACC 3′ Rev: 5′GCTGGTCCTTTGTTTGAAAGA 3′	[85]
**Co-stimulatory markers**	**CD40**	For: 5′-GCTATGGGGCTGCTTGTTGA 3′ Rev: 5′ATGGGTGGCATTGGGTCTTC 3′	[86]
	**CD80**	For: 5′CTGGGAAAAACCCCCAGAAG 3′ Rev: 5′TGACAACGATGACGACGACTG 3′	[86]
	**CD86**	For: 5′CATGGGCTTGGCAATCCTTA 3′ Rev: 5′AAATGGGCACGGCAGATATG 3′	[87]
	**CXCL1**	For: 5′GACCATGGCTGGGATTCACC 3′ Rev: 5′CCAAGGGAGCTTCAGGGTCA 3′	[88]
**Chemokines**	**CCL2**	For: 5′CCGGCTGGAGCATCCACGTGT 3′ Rev: 5′TGGGGTCAGCACAGACCTCTCTCT 3′	[89]
	**CCL5**	For: 5′ATATGGCTCGGACACCACTC 3′ Rev: 5′TCCTTCGAGTGACAAACACG 3′	[90]
	**CCL11**	For: 5′CCAGGCTCCATCCCAACTT 3′ Rev: 5′TGGTGATTCTTTTGTAGCTCTTCAGT 3′	[89]
**Internal control**	**GAPDH**	For: 5′TATGTCGTGGAGTCTACTGGT 3′ Rev: 5′GAGTTGTCATATTTCTCGT 3′	[91]

### Statistical analysis

Data acquisition for flow cytometry was performed by using BD Cell Quest (BD Bioscience). The data analysis program used was FlowJo 9.8.5 (TreeStar). The cells with >80–90% viability was selected by gating on the flow cytometer. The results from each experiment were normalized to negative control and One-way ANOVA followed by Dunnett’s Post hoc test was used to determine the statistical differences between the controls and treatments. The data are represented as means ± standard error (SE) and p values of ≤0.05 were considered to represent statistically significant differences using Prism 8.0 (GraphPad Software Inc).
